# Supplementary material for: Follow-Up of SARS-CoV-2 Antibody Levels in Belgian Nursing Home Residents and Staff Two, Four and Six Months after Primary Course BNT162b2 Vaccination
Source: Vaccines (Basel). 2024 Aug 22;12(8):951. doi: 10.3390/vaccines12080951 (PMC11359559; doi:10.3390/vaccines12080951)
Supplement: Supplementary file 1 [file vaccines-12-00951-s001.zip › vaccines-3093683-supplementary.pdf]

Supplementary Table S1. Associations between comorbidity (type/number) and SARS-CoV-2 S1RBD IgG response in nursing home residents two, four and six months post primary course vaccination.

	Condition	Mean Log10 S1RBD IgG (IU/mL)	95% Confidence Interval		p-value comorbidity[a]	p-value comorbidity*timesince vaccination[b]
**Cardiovascular disease**						
2 months	Absent	3.15	2.96	3.34		
	Present	2.96	2.81	3.10		
4 months	Absent	2.68	2.52	2.84	0.775	0.015
	Present	2.70	2.57	2.82		
6 months	Absent	2.33	2.17	2.49		
	Present	2.42	2.30	2.55		
**Diabetes mellitus**						
2 months	Absent	3.00	2.87	3.13		
	Present	3.12	2.88	3.36		
4 months	Absent	2.67	2.56	2.78	0.580	0.298
	Present	2.75	2.54	2.96		
6 months	Absent	2.39	2.28	2.50		
	Present	2.37	2.17	2.58		
**Hypertension**						
2 months	Absent	3.14	2.99	3.30		
	Present	2.88	2.71	3.05		
4 months	Absent	2.78	2.65	2.91	0.014	0.298
	Present	2.57	2.43	2.72		
6 months	Absent	2.49	2.36	2.62		
	Present	2.26	2.11	2.40		
**Severe cardiac/renal/lung disease**						
2 months	Absent	2.98	2.86	3.11		
	Present	3.27	2.98	3.56		
4 months	Absent	2.64	2.53	2.74	0.028	0.757
	Present	2.98	2.74	3.23		
6 months	Absent	2.35	2.25	2.46		
	Present	2.58	2.33	2.82		

**Immunodeficiency/immunosuppression**						
2 months	Absent	3.04	2.93	3.16	0.489	0.294
	Present	2.65	2.09	3.22		
4 months	Absent	2.69	2.59	2.79		
	Present	2.66	2.17	3.14		
6 months	Absent	2.39	2.29	2.49		
	Present	2.32	1.84	2.80		
**Cancer**						
2 months	Absent	3.02	2.90	3.14	0.713	0.757
	Present	3.26	2.58	3.94		
4 months	Absent	2.69	2.59	2.79		
	Present	2.74	2.15	3.32		
6 months	Absent	2.38	2.29	2.48		
	Present	2.41	1.83	2.99		
**Number of comorbidities**						
2 months	1	3.12	2.97	3.27	0.297	0.162
	2	2.87	2.67	3.08		
	3	2.94	2.59	3.29		
	4	3.72	1.92	5.51		
4 months	1	2.71	2.58	2.84		
	2	2.58	2.40	2.76		
	3	2.88	2.57	3.18		
	4	3.57	2.04	5.11		
6 months	1	2.42	2.29	2.55		
	2	2.31	2.13	2.48		
	3	2.39	2.09	2.70		
	4	3.21	1.71	4.72		

[a] p-values for significance of the association between the presence of the comorbidity and S1RBD IgG antibody concentrations two, four and six months post-vaccination. [b] p-values for significance of the association between the presence of the comorbidity and the slope of S1RBD IgG antibody concentrations over time. Underlined p-values are statistically significant.
